# Aneurysmal Bone Cyst of the Coracoid Process: A Case Report

**DOI:** 10.1155/cro/1355059

**Published:** 2026-05-27

**Authors:** André Castanheira, Pedro Amaro, Raquel Costa, Hugo Santos, Nuno Oliveira, Luís Pires

**Affiliations:** ^1^ Hospital Beatriz Ângelo, Loures, Portugal, hbeatrizangelo.pt

**Keywords:** aneurysmal bone cyst, biphasic bone graft substitutes, coracoid process

## Abstract

**Background:**

Aneurysmal bone cysts (ABCs) are benign yet locally aggressive lesions that can cause significant bone destruction. Involvement of the coracoid process is exceedingly rare, and the diagnosis is particularly challenging due to its atypical presentation.

**Case Presentation:**

We report the case of a 37‐year‐old male with right shoulder pain initially attributed to calcific tendinitis. Advanced imaging incidentally revealed an asymptomatic ABC of the coracoid process. Surgical management involved intralesional curettage and cavity filling with a biphasic calcium sulfate–hydroxyapatite bone graft substitute. Histopathological analysis confirmed the diagnosis of a benign ABC. Postoperative recovery was uneventful, and at 1‐year follow‐up, the patient was asymptomatic with full shoulder function. Follow‐up CT imaging showed no evidence of recurrence and resolution of the initial calcific tendinitis.

**Conclusion:**

This case highlights the importance of advanced imaging in atypical shoulder pain and demonstrates that biphasic bone graft substitutes offer a safe and effective alternative to traditional bone grafting in the treatment of ABCs, even in rare anatomical locations. Further research is needed to validate long‐term outcomes and establish standardized treatment protocols.

## 1. Introduction

An aneurysmal bone cyst (ABC) is a benign, expansile, blood‐filled cystic lesion of bone that exhibits locally aggressive behavior and a broad spectrum of skeletal involvement [1–4]. Despite its nonmalignant nature, ABC can lead to rapid bone expansion and significant destruction of osseous structures [1, 2, 4, 5].

ABCs most commonly affect the metaphysis of long bones and the posterior elements of the vertebrae, but they can occur in virtually any bone [1, 2, 4]. Among these, ABCs involving the coracoid process are exceptionally uncommon. In a review of 243 cases of scapular bone tumors, only a single case involved the coracoid process [6, 7] underscoring the rarity of this lesion in that anatomical location.

Recent molecular studies have identified recurrent rearrangements involving the USP6 gene at the 17p13.2 locus, resulting in its overexpression. These findings support the classification of ABC as a true neoplasm rather than a reactive vascular process [2, 4]. This distinction is crucial, particularly in differentiating primary ABCs from secondary forms associated with other bone tumors [2].

ABC accounts for 1%–2% of all bone neoplasms [1, 2, 4, 5] and occurs equally in males and females [2]. Most cases are diagnosed in the first two decades of life [1, 2, 4, 5]. Patients typically present with localized pain and swelling [1, 3, 5]. In more severe cases, pathologic fractures may occur, particularly in major long bones [1, 2, 4, 8]. When the spine is affected, neurological symptoms due to spinal cord or nerve root compression may be the first signs of disease [2, 4].

Diagnosis of ABC involves a combination of clinical evaluation, radiologic imaging, and histological analysis to distinguish between primary and secondary forms [1, 2, 5]. Radiographs and computed tomography (CT) help assess the extent of bone involvement and detect associated fractures [1, 2], whereas magnetic resonance imaging (MRI) is useful for evaluating soft tissue and intramedullary extension [1].

Radiographically, ABCs appear as eccentric, radiolucent lesions with expansile bone remodeling and a multilocular appearance due to internal septation. CT typically shows a well‐defined lytic lesion surrounded by a thin rim of reactive bone. While fluid‐fluid levels may be occasionally detected on CT, MRI is superior for identifying these characteristic features [1, 2, 4, 5].

Tumors of the coracoid process are especially prone to misdiagnosis, as their clinical presentation often mimics more common, non‐neoplastic shoulder pathologies such as instability, impingement syndrome, tendinitis, and adhesive capsulitis (frozen shoulder) [6]. Furthermore, standard anteroposterior shoulder radiographs may fail to detect lesions in the coracoid process in up to 10% of cases, making diagnosis even more challenging [6].

Histologically, ABCs consist of cavernous or slit‐like blood‐filled spaces separated by fibrous septa. These septa contain spindle cells, inflammatory cells, and osteoclast‐like multinucleated giant cells, often clustered around hemorrhagic spaces [1, 2, 4, 5].

Treatment goals include pain control, stabilization, and prevention of pathologic fractures [2, 5]. Surgical curettage with or without bone grafting remains the mainstay of treatment [1, 2, 4, 5]. Although en bloc resection has the lowest recurrence rate, it is rarely performed due to increased risk of functional impairment and morbidity [1, 2, 4, 5].

## 2. Case Presentation

**Figure 1:**
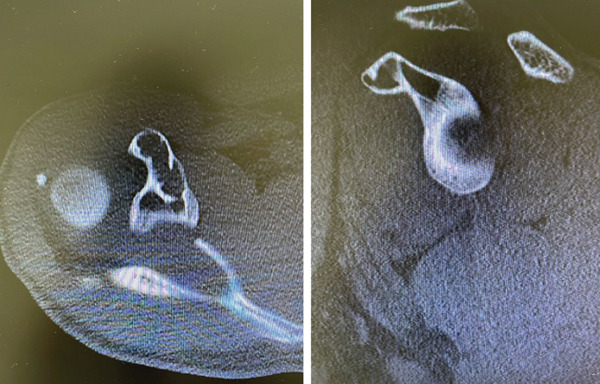
Preoperative computed tomography (CT) scan of the right shoulder demonstrating a well‐defined, expansile osteolytic lesion located in the coracoid process. The lesion presents with cortical thinning and multiloculated appearance, without evidence of cortical breach or periosteal reaction. These features are consistent with an aneurysmal bone cyst.

**Figure 2:**
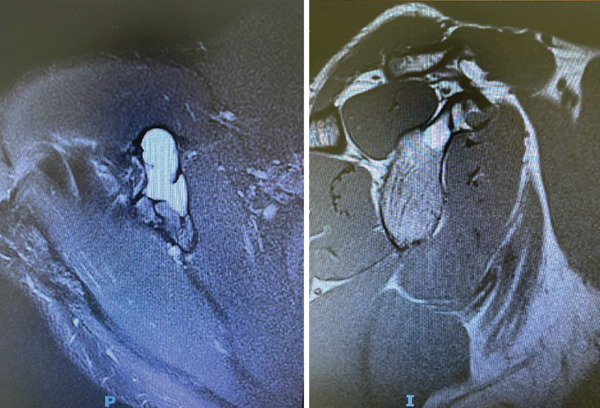
Preoperative magnetic resonance imaging (MRI) of the right shoulder showing a cystic lesion in the coracoid process. The lesion demonstrates heterogeneous signal intensity with multiple internal septations and fluid‐fluid levels, characteristic of an aneurysmal bone cyst. No significant soft tissue extension or surrounding edema is observed.

We present the case of a 37‐year‐old male with no significant medical history who reported insidious, dull right shoulder pain persisting for several weeks. Initial evaluation at a private healthcare facility included plain radiographs, CT, and MRI. Imaging revealed calcific tendinitis of the supraspinatus tendon, for which he was treated with NSAIDs and physical therapy. Incidentally, an ABC of the coracoid process was identified, prompting referral to our shoulder surgery clinic (Figures 1 and 2).

The patient reported partial symptom relief following conservative treatment. On physical examination, no swelling or skin changes were observed. Active range of motion was preserved but painful, particularly during abduction between 60° and 120°. Forward elevation reached 160°, external rotation 60°, and internal rotation to L3, all limited by pain. Strength testing revealed mild weakness, with abduction graded at 4/5 and external rotation at 4+/5. Impingement signs, including Neer and Hawkins tests, were positive. The pain was severe enough to interfere with daily activities, including overhead use and sleep.

Surgical management was recommended. The procedure included cyst curettage followed by filling with a biphasic calcium sulfate–hydroxyapatite bone graft substitute. After detailed discussion of risks and benefits, informed consent was obtained.

**Figure 3:**
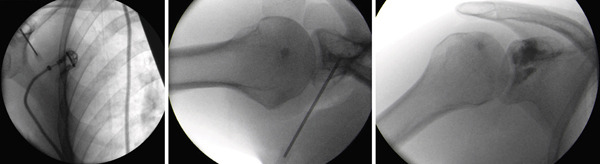
Intraoperative fluoroscopic images demonstrating the surgical management of the lesion. After intralesional curettage of the cyst, the residual cavity was filled with a biphasic calcium sulfate–hydroxyapatite bone graft substitute. Adequate filling of the defect and preservation of the coracoid structure are confirmed under fluoroscopic guidance.

The patient was placed in the beach chair position. A mini deltopectoral approach with superior extension was used to access the coracoid. Under fluoroscopic guidance, the anterosuperior cyst wall was drilled. Cystic fluid was aspirated, followed by curettage of the lesion. Solid tissue obtained during curettage was submitted for histopathological evaluation. The cavity was then filled with 5 cc of biphasic bone graft substitute, ensuring adequate filling under fluoroscopic control (Figure 3). The surgical site was closed in layers.

Postoperative recovery was uneventful. Follow‐ups were conducted at 1, 2, and 8 weeks, and at 1 and 2 years postsurgery. The patient was prescribed analgesics for 1 week and started physiotherapy after suture removal at 2 weeks. At 8 weeks, histopathology showed no cellular atypia, excluding malignancy. A control CT revealed a central sclerotic area corresponding to the treated ABC, measuring 21 × 16 mm, without signs of cortical disruption or aggressive behavior. Rotator cuff calcification was noted, with a 12‐mm Type I arc‐shaped macrocalcification in the supraspinatus tendon.

**Figure 4:**
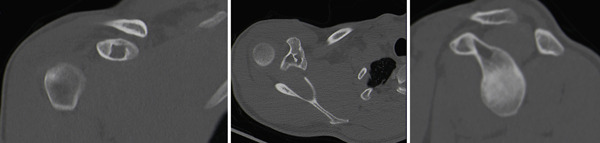
One‐year postoperative CT scan of the right shoulder demonstrating a stable residual cystic cavity within the coracoid process. There is no evidence of lesion progression, cortical disruption, or recurrence. The previously noted central sclerotic area has resolved, and overall bone architecture is preserved.

At 1‐year follow‐up, the patient was asymptomatic. Shoulder range of motion was fully restored, and surgical healing was satisfactory. The QuickDASH score was 0. A control CT showed that the previously dense central focus in the coracoid was no longer visible. The residual cystic cavity remained stable at 12 × 21 × 19 mm, without signs of aggression. Additionally, the supraspinatus tendon calcification had been resolved (Figure 4).

At 2‐year follow‐up, the patient remained asymptomatic, with no evidence of recurrence on plain radiographs. In the absence of clinical or radiographic suspicion, additional CT imaging was not performed to avoid unnecessary radiation exposure.

## 3. Discussion

In the present case, the patient′s symptoms were most consistent with calcific tendinitis, as suggested by the clinical presentation and imaging findings, including pain during the painful arc and partial response to NSAIDs and physiotherapy. Calcific tendinitis is typically a self‐limiting condition, often resolving spontaneously or with conservative treatment. The ABC of the coracoid was considered an incidental finding, as it did not correlate clearly with the pain pattern or physical examination. This highlights the critical role of advanced imaging modalities, such as CT and MRI, in the comprehensive assessment of atypical or unexplained shoulder pain [2, 4, 5].

The indication for surgical treatment of the ABC was based not on pain, but on its structural characteristics. ABCs are locally aggressive lesions with potential for progressive expansion, cortical thinning, and risk of pathological fracture [1, 2, 4, 5], which occur in approximately 8% of cases [8], particularly in anatomically critical regions such as the coracoid process [6, 7]. Given these risks, surgical management was indicated despite the absence of clear symptom attribution.

Open curettage followed by bone grafting remains the standard therapeutic approach [1, 2, 4, 5]. While recurrence rates after standard curettage can reach 30%, the use of high‐speed burrs and adjuvant therapies has been shown to reduce this rate to around 15% [3].

Emerging evidence supports the use of bone substitutes as a reliable option for cavity filling after cyst decompression. Although recurrence rates appear similar between bone grafting and cementation techniques, the literature does not clearly favor one approach over the other [4, 9]. Importantly, there remains a paucity of large‐scale cohort studies examining the use of bone substitutes in ABC treatment [9]. Nevertheless, smaller studies have suggested that curettage followed by cementation may result in lower recurrence rates compared to bone grafting [10].

En bloc excision provides the highest rate of local disease control (95%–100%) but is associated with considerable morbidity, including postoperative pain, limb length discrepancy, muscle weakness, and decreased range of motion [4, 5]. As such, this approach is typically reserved for recurrent, refractory cases or for lesions in locations where resection would not significantly compromise function [4].

Given this background, our choice of intralesional curettage followed by filling the cavity with a biphasic bone graft substitute offered a balanced approach, minimizing surgical morbidity without compromising efficacy. At 1‐year follow‐up, control CT imaging demonstrated a stable residual cystic cavity, with no signs of recurrence, and the patient was asymptomatic. Given the self‐limiting nature of calcific supraspinatus tendinopathy and its well‐established response to nonsteroidal anti‐inflammatory drugs and physiotherapy, symptom resolution is most likely attributable to conservative management and the natural course of the disease, rather than the surgical treatment of the incidental ABC. At 2‐year follow‐up, plain radiographs confirmed continued stability.

Although late recurrences can occur, most are observed within the first 2 years following treatment. De Silva et al. reported that 90% of recurrences occurred within the first year [8]. Therefore, regular postoperative imaging, particularly within the first year, is essential for early detection of recurrence and appropriate intervention.

## 4. Conclusion

This case demonstrates that the use of a biphasic bone graft substitute for treating ABCs in rare anatomical locations, such as the coracoid process, is a safe and effective alternative to conventional curettage and bone grafting. It offers reduced operative morbidity and maintains favorable clinical and radiologic outcomes. Further prospective studies with larger cohorts are necessary to validate the long‐term efficacy of this technique and define its place in standard treatment protocols.

## Funding

No funding was received for this manuscript.

## Conflicts of Interest

The authors declare no conflicts of interest.

## Data Availability

The data that support the findings of this study are available from the corresponding author upon reasonable request.
